# Supplementary material for: Psycho-oncologists’ roles, tasks, and needs regarding requests for assisted suicide – a qualitative interview study on current experiences and future directions
Source: Support Care Cancer. 2026 Apr 16;34(5):433. doi: 10.1007/s00520-026-10656-4 (PMC13083415; doi:10.1007/s00520-026-10656-4)
Supplement: Supplementary file 3 — (PDF 128 KB) [file 520_2026_10656_MOESM3_ESM.pdf]

Supporting Information 3 | Demographic Online Questionnaire for Interview Participants (English translation)

Note: This is an English translation. The original questionnaire was in German.

Please check the appropriate box(es):

1. What gender do you identify as? *(Multiple answers possible)*

   ☐ Female
   ☐ Male
   ☐ Non-binary/diverse
   ☐ Other or prefer not to say

2. Which age group do you belong to?

   ☐ Under 30 years
   ☐ 31 to 40 years
   ☐ 41 to 50 years
   ☐ 51 to 60 years
   ☐ Over 60 years
   ☐ Prefer not to say

3. In which federal state do you work?

   ☐ Baden-Württemberg
   ☐ Bavaria
   ☐ Berlin
   ☐ Brandenburg
   ☐ Bremen
   ☐ Hamburg
   ☐ Hessen
   ☐ Mecklenburg-Western Pomerania
   ☐ Lower Saxony
   ☐ North Rhine-Westphalia
   ☐ Rhineland-Palatinate
   ☐ Saarland
   ☐ Saxony
   ☐ Saxony -Anhalt
   ☐ Schleswig-Holstein
   ☐ Thuringia
   ☐ Prefer not to say

4. In which setting do you currently work as a psycho-oncologist? *(Multiple answers possible)*

   ☐ Hospital: Outpatient care
   ☐ Hospital: Inpatient care (e.g., ward psychologist, liaison service)
   ☐ Hospital: Consultation service
   ☐ Cancer counseling center
   ☐ Psychotherapy practice
   ☐ Inpatient palliative care
   ☐ Outpatient palliative care
   ☐ Rehabilitation hospital
   ☐ Prefer not to say

5. What is your professional background?
   (Multiple answers possible)

   ☐ Clinical psychologist*
   ☐ Psychologist
   ☐ Physician trained in psychotherapy**
   ☐ Physician
   ☐ Educator
   ☐ Prefer not to say
   ☐ Other professional background (please specify in the comment field):
   ____

6. How many years of work experience do you have in psycho-oncology?

   ☐ Less than 1 year
   ☐ 1 to less than 5 years
   ☐ 5–10 years
   ☐ 11–15 years
   ☐ 16-20 years
   ☐ More than 20 years
   ☐ Prefer not to say

7. With how many of your psycho-oncological patients did you discuss assisted suicide? *(An approximate estimate is sufficient.)*

   ☐ 0
   ☐ 1-5
   ☐ 6-10
   ☐ 11-20
   ☐ 21-50
   ☐ More than 50
   ☐ Prefer not to say

<u>Notes:</u>
* In German: ‚psychologische:r Psychotherapeut:in', i.e., a licensed clinical psychologist
** In German: „ärztliche:r Psychotherapeut:in", i.e., a physician with additional training and a license to provide psychotherapy
