# Supplementary material for: Psycho-oncologists’ roles, tasks, and needs regarding requests for assisted suicide – a qualitative interview study on current experiences and future directions
Source: Support Care Cancer. 2026 Apr 16;34(5):433. doi: 10.1007/s00520-026-10656-4 (PMC13083415; doi:10.1007/s00520-026-10656-4)
Supplement: Supplementary file 5 — (PDF 178 KB) [file 520_2026_10656_MOESM5_ESM.pdf]

Supporting Information 5 | Themes and corresponding codes

Notes: These results are based on 12 interviews and should be interpreted as purely exploratory. Psych = psycho-oncologist(s), pat = patient(s), HCPs = healthcare providers. Code IDs are given in brackets after each code.

Theme 1: Psycho-oncologists involvement in AS-related tasks must be voluntary

- AS tasks must be based on the voluntariness of psych (B1)

Theme 2: Psycho-oncologists mainly react to AS being brought up, some also initiate AS conversations

- AS conversations are mostly initiated by pat, rarely by psychologists (A2.1)
  - Psych hear the topic, when pat bring up AS (A2.1.1)
  - Psych proactively raised the topic of AS with pat (A2.1.2)

Theme 3: Psycho-oncologists are and should be open, non-judgmental, dependable and longitudinal conversation partners

- Psych are open conversation partners (A1.1)
- Psych are longitudinal supporters (A1.2)
- Psych talk about AS, but are not realizing AS (A1.4)
- Psych stay in contact, if a pat is already registered for AS (A2.11)
- Psych should be open conversation partners (B2.1)
- Psych could be involved early in someone's AS considerations (B2.8)

Theme 4: Psycho-oncologists do and should guide patients through AS-related decision-making and planning

- Psych explore AS wishes (A2.2)
- Psych provide neutral information about AS (A2.5)
- Psych discuss alternatives to AS (A2.6)
- Psych support pat during AS planning (A2.8)
- Psych should explore AS wishes (B2.3)
- Psych should provide neutral information about AS (B2.4)
- Psych should discuss alternatives to AS (B2.5)
- Psych could address spiritual and religious concerns (B2.8)
- Psych should support pat during AS planning (B3.2, controversially perceived)

Theme 5: Psycho-oncologists do and should coordinate AS-related care between patients, relatives, and other professionals

- Psych are "bridge builders" (A1.3)
  - Psych involve relatives (A1.3.1)
  - Psych collaborate with HCPs and others (A1.3.2)
- Psych refer pat to additional support structures (A2.7)
- Psych support pat relatives during and/or after AS process (A3.1)
- Psych should be "bridge builders" (B2.2)
  - Psych should involve relatives (B2.2.1)
  - Psych should cooperate with other professionals (B2.2.2)
- Psych should refer pat to additional support structures (B2.6)
- Psych could offer supervision on AS to other health care professionals (B2.9)
- Support of pat relatives during and/or after AS process (B3.3, controversially perceived)
  - Psych should support pat relatives (B3.3.1)
  - Psych should not be responsible for pat relatives (B.3.3.2)

Theme 6: Psycho-oncologists offer psychotherapeutic services related to AS consideration

- Psych explore, document, and report suicidality (A2.3)
- Psych offer psychotherapeutic interventions (A2.10)
- Psych should offer psychotherapeutic interventions (B2.7)

Theme 7: Psycho-oncologists consider legal and ethical issues related to AS

- Psych informally explore decision-making capacity (A2.4)
- Psych focus on balancing respect for pat autonomy and protecting life (A2.9)

Theme 8: Psycho-oncologists should (not) be involved in AS-related evaluation and granting or denying of access

- Assessment of decision-making capacity (B3.1, controversially perceived)
  - Psych should assess decision-making capacity (B3.1.1)
  - Psych should not assess decision-making capacity (B3.1.2)
- Mandatory psychological counseling regarding AS (B3.4, controversially perceived)
  - Psych should provide mandatory counseling on AS (B.3.4.1)
  - Psychologists reject mandatory counseling on AS (B.3.4.2)

Theme 9: Psycho-oncologists should (not) be involved in AS-realization

- Involvement during realization of AS (B3.5, controversially perceived)

  - Psych could support pat throughout the entire AS process, including realization (B3.5.1)
  - Psych should not be involved during realization of AS (B3.5.2)

Theme 10a: AS conversations require specific competencies

- AS conversations partners should have appropriate and sufficient competences regarding AS (B4.1)
- Other professionals are also suitable for AS talks (B4.2)
- Palliative care psychologists may be especially qualified for AS work (B4.3)

Theme 10b: Psycho-oncologists' knowledge and perceived competence regarding AS vary

- Psych feel inadequately informed and unprepared regarding AS (A4.1)
- Psych feel insecure handling AS requests from certain vulnerable groups (A4.2)
- Psych feel well-prepared for aspects of AS (A4.3)

Theme 10c: Psycho-oncologists already engage in, but need more AS-related education

- Psych organized AS-related professional events (A3.2)
- Psych actively seek education and training on AS for themselves (A3.3)
- Psych need specific training on AS (C1.1)
- Psych need practical workshops on AS communication skills (C1.2)
- Psych need clarity about their personal attitudes toward AS (C1.3)

Theme 11: Psycho-oncologists need evidence-based guidelines and tools related to AS

- Psych need evidence-based guidelines and tools (C2)

Theme 12a: Psycho-oncologists' AS-related roles and tasks are influenced by the institutional and legal context

- AS tasks are highly setting- and context-dependent (A5.1)
- Guidelines and policies are unclear or lacking (A5.2)
- Institutional policies restrict AS work (A5.3)
- The extent of collegial exchange varies (A5.4)
  - Collegial exchange on AS is limited (A5.4.1)
  - Collegial exchange on AS occurs (A.5.4.2)
- Suitable spaces are lacking (A5.5)
- There is a lack of financial reimbursement for some AS-related tasks (A5.6)

Theme 12b: Psycho-oncologists need an appropriate institutional situation

- Institutional policies should not restrict open AS conversations (C4.1)
- Psych need a well-organized team with good communication (C4.2)
- Psych need confidential spaces for AS conversations (C4.3)
- Psych need adequate time for pat interactions (C4.4)
- Psych need clear referral pathways and contact persons responsible for AS (C4.6)
- There should be AS-related information sessions for pat (C4.7)

Theme 12c: Psycho-oncologists need AS-related (healthcare) system changes and legal clarity

- AS-related tasks must be eligible for financial reimbursement (C4.5)
- Psychologists need clear legal regulations (C4.8)

Theme 13: Psycho-oncologists need societal acceptance of AS

- Psych need societal acceptance of AS (C5)

Theme 14: Psycho-oncologists need to also care for themselves

- Psych need supervision and collegial exchange (C2.1)
- Psych need self-care (C2.2)
